# Co-colonization of different species harboring KPC or NDM carbapenemase in the same host gut: insight of resistance evolution by horizontal gene transfer

**DOI:** 10.3389/fmicb.2024.1416454

**Published:** 2024-06-14

**Authors:** Jingshu Ji, Yufeng Zhu, Feng Zhao, Jingjing Zhang, Bingyan Yao, Mingli Zhu, Yunsong Yu, Jun Zhang, Ying Fu

**Affiliations:** ^1^Department of Clinical Laboratory, Sir Run Run Shaw Hospital, School of Medicine, Zhejiang University, Hangzhou, Zhejiang, China; ^2^Key Laboratory of Precision Medicine in Diagnosis and Monitoring Research of Zhejiang Province, Hangzhou, Zhejiang, China; ^3^Department of Open Laboratory Medicine, Hangzhou Xixi Hospital, School of Medicine, Zhejiang University, Hangzhou, Zhejiang, China; ^4^Department of Infectious Diseases, Sir Run Run Shaw Hospital, School of Medicine, Zhejiang University, Hangzhou, Zhejiang, China; ^5^Key Laboratory of Microbial Technology and Bioinformatics of Zhejiang Province, Hangzhou, Zhejiang, China; ^6^Regional Medical Center for National Institute of Respiratory Diseases, Sir Run Run Shaw Hospital, School of Medicine, Zhejiang University, Hangzhou, Zhejiang, China

**Keywords:** carbapenem-resistant Enterobacteriales, KPC, NDM, horizontal gene transfer, interspecies, host gut

## Abstract

**Introduction:**

The dissemination of carbapenem-resistant Enterobacteriales (CRE) in nosocomial settings is primarily associated with the horizontal transfer of plasmids. However, limited research has focused on the in-host transferability of carbapenem resistance. In this study, ten isolates were collected from gut specimens of five individuals, each hosting two different species, including *Escherichia coli*, *Klebsiella pneumoniae*, *Klebsiella aerogenes*, *Enterobacter cloacae*, or *Citrobacter koseri*.

**Methods:**

Species identification and antimicrobial susceptibility were determined by MALDI-TOF MS and broth microdilution method. Carbapenemase genes were detected and localized using PCR, S1-PFGE and southern blot. The transferability of carbapenemase genes between species was investigated through filter mating experiments, and the genetic contexts of the plasmids were analyzed using whole genome sequencing.

**Results and discussion:**

Our results revealed that each of the ten isolates harbored a carbapenemase gene, including *bla*_NDM-5_, *bla*_NDM-1_, or *bla*_KPC-2_, on a plasmid. Five different plasmids were successfully transferred to recipient cells of *E. coli, K. pneumoniae* or *A. baumannii* by transconjugation. The genetic contexts of the carbapenemase gene were remarkably similar between the two CRE isolates from each individual. This study highlights the potential for interspecies plasmid transmission in human gut, emphasizing the colonization of CRE as a significant risk factor for the dissemination of carbapenemase genes within the host. These findings underscore the need for appropriate intestinal CRE screening and colonization prevention.

## Introduction

Antibiotic resistance of bacteria has become a global concern, particularly in clinical settings (Laxminarayan et al., 2013). Carbapenems are often considered as a last resort for treating multidrug-resistant bacteria, alongside polymyxin and tigecycline (Brink, 2019). Among the various mechanisms of carbapenem resistance, the production of carbapenemase is a significant factor (Mancuso et al., 2021). The spread of carbapenemase between different pathogens is primarily associated with horizontal gene transfer (HGT) (Partridge et al., 2018; San Millan, 2018). Conjugation, mediated by transferable elements such as plasmids and integrative conjugative elements (ICE), is a major mechanism of HGT, surpassing transduction and transformation (Halary et al., 2010; Partridge et al., 2018). Consequently, the transfer of carbapenemase-producing plasmids between pathogens could exacerbate the antibiotic resistance situation and contribute to the global distribution of carbapenem-resistant Enterobacteriales (CRE).

The human gut serves as a natural reservoir for a diverse microbial population, including important pathogens with varying resistances and relevant genes, making it an ideal environment for interspecies HGT (McInnes et al., 2020). Given that gut microbiota act as a defense against pathogen invasion, disruption of this protection could lead to gut-derived infections in critically ill patients (Wang et al., 2019). The use of broad-spectrum antibiotics is known as an important risk of microbiota disruption and CRE colonization, such as carbapenems, cephalosporins, ciprofloxacin and so on (Kim et al., 2017). As we have known, bacteria need nutrients to grow and colonize in gut, some of its nutrient metabolites even can inhibit pathogen growth (Ng et al., 2013). However, the killing of gut microbiota by broad-spectrum antibiotics, not only enriches the nutrients for CRE to grow, but also reduces the inhibitory metabolites, causing the CRE expansion and colonization (Reed and Theriot, 2021; Yip et al., 2023). Common opportunistic pathogens in the human gut microbiota include Enterobacteriales such as *Escherichia coli* and *Klebsiella pneumoniae*. Colonization of multidrug-resistant *K. pneumoniae* in patients’ gut can lead to transmission in the intensive care unit, with approximately 50% of infections originating from the patients’ own microbiota (Gorrie et al., 2017). Previous studies have reported the transfer of carbapenem-resistant Enterobacteriales between patients, which is significant in healthcare-associated infections (David et al., 2019). However, the *in vivo* transmission of carbapenem-resistant genes between gut microbes are not fully understood.

Therefore, we conducted this study to assess the colonization of carbapenem-resistant bacteria in patients’ gut in our hospital and to investigate their potential *in vivo* transmission pathways.

## Materials and methods

### Project design, bacterial isolation and antimicrobial susceptibility testing

A project was initiated to monitor the colonization of carbapenem-resistant Enterobacterales in the human gut and to assess the risk of secondary infections in hospitalized patients. The project was conducted in accordance with the ethical approval granted by Sir Run Run Shaw Hospital under the reference number 20200831-36, spanning from 2018 to 2021.

CRE species were initially isolated from stool samples using Chromatic CRE selective plates (Liofilchem® Chromatic™, Italy) following standard manufacturing protocols. Colonies grown on the plates were then identified using Matrix Assisted Laser Desorption Ionization Time-of-Flight mass spectrometry (MALDI-TOF MS, bioMérieux, France) and tested for susceptibility to meropenem, imipenem, and ertapenem. Isolates identified as Enterobacterales species and confirmed to be resistant to any of the three carbapenems were subjected to antimicrobial susceptibility testing (AST) using the VITEK2 compact system (bioMérieux, France) for 16 antimicrobial agents (ampicillin; ampicillin/sulbactam; piperacillin/tazobactam; ceftazidime; cefotetan; ceftriaxone; cefepime; imipenem; ertapenem; aztreonam; ciprofloxacin; gentamicin; amikacin; levofloxacin; sulfamethoxazole/trimethoprim; tobramycin), and three others (tigecycline; colistin; ceftazidime/avibactam) were tested using the broth microdilution method as per CLSI M07Ed11 guidelines (CLSI, 2018). The MIC interpretation followed the CLSI M100 (30th edition) (CLSI, 2020) breakpoints, except for tigecycline, for which the breakpoints and interpretive criteria of EUCAST (Version 11.0, 2021) were applied (EUCAST, 2021). Quality control strains included *E. coli* ATCC25922 and *Pseudomonas aeruginosa* ATCC 27853.

The presence of carbapenemase in CRE was determined using NG-Test® CARBA 5 (Hardy Diagnostics, France) and confirmed by PCR amplification followed by sequencing (Du et al., 2013). Patients with two CRE species were included, and the CRE isolates were collected for further analysis.

### Plasmid analysis and southern hybridization

The bacterial isolates were embedded in agarose plugs containing total DNAs, and then treated with S1 nuclease. Plasmids were separated using the CHEF-Mapper XA PFGE system (Bio-Rad, United States) (Hunter et al., 2005). The gel was stained with GelRed (Biotium, United States) and visualized under 254 nm UV light. Subsequently, the DNAs on the gel were transferred to a nylon membrane (Millipore, United States) under pressure, hybridized with digoxigenin-labeled *bla*_NDM_ or *bla*_KPC_ probes, and detected using the NBT/BCIP color detection kit (Roche Applied Sciences, Germany) (Chen et al., 2011).

### Filter mating experiment

To assess the transferability of plasmids carrying carbapenem-resistant genes through conjugation, a filter mating experiment was conducted between donor cells containing the carbapenem-resistant gene on plasmid and recipient cells of different species. Four recipient isolates from three species were selected: *E. coli* J53 (Sodium azide resistant, NaN_3_^R^), *E. coli* EC600 (Rifampicin resistant, Rif^R^), *Acinetobacter baumannii* ATCC17978 (Rifampicin resistant, Rif^R^), and *K. pneumoniae* ATCC13883 (Rifampicin resistant, Rif^R^) (Supplementary Table S1). Each recipient strain was mated with the donor strain, except when both of them were of the same species. The mating experiment was performed on a 0.22 μm filter membrane cultured on antibiotic-free Mueller-Hinton agar medium. After 18 h of incubation at 37°C, the bacterial mixture was transferred to Mueller-Hinton agar medium supplemented with specific antibiotics as listed in Supplementary Table S1. The transconjugants were then confirmed by PCR for the presence of carbapenemase genes. Antimicrobial susceptibility testing of the transconjugants was conducted using the methods described above.

### Whole genome sequencing and plasmid analysis

Genomic DNAs of all the isolates were extracted using the QIAamp DNA MiniKit (Qiagen, New York, United States) according to the manufacturer’s instructions. Subsequently, the DNA samples were sequenced using the Illumina-Hiseq X-10 (Illumina Inc., San Diego, CA) and/or the MinION platform (Nanopore, Oxford, United Kingdom). All the 10 CRE isolates were sequenced by the Illumina-Hiseq X-10 (Illumina Inc., San Diego, CA), while only 6 isolates (Eco13188, Eco20779, Eco20155, Kpn20156, Kpn20795 and Kpn40) were sequenced by the MinION platform (Nanopore, Oxford, UK). The sequenced reads were assembled using LC Genomic Workbench (CLC Bio 10.0) and Unicycler software. Gene prediction and annotation were performed using prokka. Plasmid maps were generated and compared using Proksee^1^ (Grant et al., 2023). The whole genome sequences were submitted to the Center for Genomic Epidemiology website^2^ for analysis of plasmid types, virulence genes, and resistance genes. The genome sequences of isolates Eco13188, Eco20779, Eco20155, Kpn20156, Kpn20795 and Kpn40 have been deposited in NCBI under the BioProject number PRJNA809193.

## Results

### Basic information of the patients

During the course of the project between July and September 2021, five inpatients (referred to as P1 to P5) were included, each of whom harbored two distinct CRE species simultaneously (Table 1). The patients’ ages ranged from 30 to 88 years, with an average age of 60.8 years. They were admitted to the Hematology department (2 patients), Infectious Disease department (2 patients), and Gastroenterology department (1 patient). All patients were afflicted with severe illnesses, such as hematological malignancies, septicemia, and hepatic failure.

**Table 1:** Basic information of patient confronting CRE colonization.

Patient no.	Gender	Age	Ward	Diagnosis	Species	Strain No.	Date	Carbapenemase type
P1	M	48	Hematology	Multiple myeloma	*E. coli*	Eco13188	2021.7.27	NDM-5
					*C. koseri*	Cko20222	2021.7.30	NDM-5
P2	M	88	Infectious disease	Non-Hodgkin’s lymphoma and pyohemia	*E. coli*	Eco20779	2021.9.10	KPC-2
					*K. aerogenes*	Eae20780	2021.9.10	KPC-2
P3	F	58	Infectious disease	Diabetes and pyohemia	*E. coli*	Eco20155	2021.8.6	KPC-2
					*K. pneumoniae*	Kpn20156	2021.8.6	KPC-2
P4	M	30	Hematology	Myelodysplastic syndrome	*K. pneumoniae*	Kpn20795	2021.9.12	NDM-1
					*E. cloacae*	Ecl20823	2021.9.12	NDM-1
P5	M	80	Gastroenterology	Hepatic failure	*K. pneumoniae*	Kpn40	2021.9.19	NDM-5
					*E. coli*	Eco41	2021.9.19	NDM-5

### Species identification and antimicrobial susceptibility test of CREs

A total of 10 CRE isolates were collected and identified. The antimicrobial susceptibility testing of the 10 isolates is summarized in Table 2 (Donor part), indicating resistance to imipenem, ertapenem, ampicillin, ampicillin/sulbactam, and ceftriaxone, while showing susceptibility to tigecycline, colistin, and amikacin. Subsequent testing using NG-Test® CARBA 5 and PCR sequencing confirmed the production of NDM-1 carbapenemase in isolates from P4, NDM-5 carbapenemase in isolates from P1 and P5, and KPC-2 carbapenemase in isolates from P2 and P3 (Table 1).

**Table 2:** Antimicrobial susceptibility testing of both donors, recipients and transconjugants (μg/mL).

Strain	Strain no.	VITEK result																Broth dilution method		
		AMP	SAM	TZP	CAZ	CTT	CRO	FEP	IMP	ERP	ATM	CIP	GEN	AMK	LEV	SXT	TOB	TGC	CO	CZA
Donor	Eco13188	**≥32**	**≥32**	**≥128**	**≥64**	**≥64**	**≥64**	**≥64**	**≥16**	**≥8**	**≥64**	**≥4**	**≥16**	≤2	**≥8**	**≥320**	**≥16**	0.5	1	**>128**
	Cko20222	ND	ND	ND	**≥64**	**≥64**	**≥64**	**16**	**4**	**≥8**	2	**1**	**≥16**	≤2	1	≤20	**≥16**	2	2	**128**
	Eco20779	**≥32**	**≥32**	**≥128**	**16**	**32**	**≥64**	8	**≥16**	**≥8**	**≥64**	**≥4**	**≥16**	16	**≥8**	**≥320**	**≥16**	0.25	2	≤0.125
	Eae20780	ND	ND	**≥128**	**≥64**	**≥64**	**≥64**	**≥64**	**≥16**	**≥8**	**≥64**	**≥4**	≤1	16	4	≤20	8	4	1	2
	Eco20155	**≥32**	**≥32**	64	4	≤4	**8**	2	**4**	**4**	**16**	**≥4**	≤1	≤2	**≥8**	≤20	≤1	1	1	≤0.25
	Kpn20156	**≥32**	**≥32**	**≥128**	**≥64**	**≥64**	**≥64**	**≥64**	**≥16**	**≥8**	**≥64**	**≥4**	**≥16**	≤2	**≥8**	≤20	8	2	1	1
	Kpn20795	**≥32**	**≥32**	64	**≥64**	≤4	**16**	2	**≥16**	**4**	≤1	≤0.25	≤1	≤2	≤0.25	≤20	≤1	0.5	2	**>128**
	Ecl20823	ND	ND	64	**≥64**	**≥64**	**≥64**	**32**	**≥16**	**≥8**	≤1	0.5	≤1	≤2	1	**≥320**	≤1	4	1	**>128**
	Kpn40	**≥32**	**≥32**	**≥128**	**≥64**	**≥64**	**≥64**	**32**	**≥16**	**≥8**	≤1	≤0.25	≤1	≤2	≤0.25	≤20	≤1	1	1	**>128**
	Eco41	**≥32**	**≥32**	**≥128**	**≥64**	**≥64**	**≥64**	**≥64**	**≥16**	**4**	≤1	≤0.25	≤1	≤2	≤0.25	≤20	≤1	0.25	1	**>128**
Recipient	*E. coli* J53	4	≤2	≤4	≤1	≤4	≤1	≤1	≤1	≤0.5	≤1	≤0.25	≤1	≤2	≤0.25	≤20	≤1	0.25	2	ND
	*E. coli* EC600	8	4	≤4	≤1	≤4	≤1	≤1	≤1	≤0.5	≤1	≤0.25	≤1	≤2	0.5	≤20	≤1	0.125	1	ND
	*K. pneumoniae* ATCC13883	**≥32**	4	≤4	≤1	≤4	≤1	≤1	2	≤0.5	≤1	≤0.25	≤1	≤2	≤0.25	≤20	≤1	0.25	2	ND
	*A. baumannii* ATCC17978	ND	≤2	ND	4	ND	8	2	≤1	ND	ND	≤0.25	≤1	4	≤0.25	**160**	≤1	0.25	4	ND
Transconjugant	*K. pneumoniae* ATCC13883 (pNDM-13188)	**≥32**	**≥32**	**≥128**	**≥64**	**≥64**	**≥64**	**≥64**	**≥16**	**≥8**	≤1	≤0.25	≤1	≤2	≤0.25	≤20	≤1	0.25	1	**>128**
	*K. pneumoniae* ATCC13883 (pKPC-20779)	**≥32**	**≥32**	≥128	**16**	**16**	**≥64**	**16**	**≥16**	**≥8**	**≥64**	**1**	≤1	8	1	**80**	8	0.125	1	≤0.125
	*A. baumannii* ATCC17978 (pKPC-20779)	ND	**≥32**	ND	**32**	ND	**≥64**	**≥64**	**≥16**	ND	ND	0.5	≤1	4	≤0.25	**160**	8	0.25	2	ND
	*E. coli* J53 (pKPC-20780)	**≥32**	**≥32**	**≥128**	**16**	**16**	**≥64**	**32**	**≥16**	**≥8**	**≥64**	**1**	≤1	16	1	**80**	**≥16**	0.25	2	≤0.125
	*E. coli* EC600(pKPC-20780)	**≥32**	**≥32**	**≥128**	**≥64**	**≥64**	**≥64**	**≥64**	**≥16**	**≥8**	**≥64**	**≥4**	≤1	16	4	≤20	**≥16**	0.125	1	≤0.125
	*K. pneumoniae* ATCC13883 (pKPC-20780)	**≥32**	**≥32**	**≥128**	**16**	8	**≥64**	**16**	**≥16**	**≥8**	**≥64**	**1**	≤1	16	1	**80**	8	0.125	1	≤0.125
	*A.baumannii* ATCC17978 (pKPC-20780)	ND	**≥32**	ND	**≥64**	ND	**≥64**	**≥64**	**≥16**	ND	ND	0.5	≤1	4	0.5	**160**	8	0.125	2	ND
	*E. coli* J53 (pNDM-20795)	**≥32**	**≥32**	64	**≥64**	**16**	**32**	2	**8**	2	≤1	≤0.25	≤1	≤2	≤0.25	≤20	≤1	0.125	2	**>128**
	*E. coli* EC600 (pNDM-20795)	**≥32**	**≥32**	64	**≥64**	**16**	**≥64**	2	**8**	2	≤1	≤0.25	≤1	≤2	0.5	≤20	≤1	0.25	1	**>128**
	*A. baumannii* ATCC17978 (pNDM-20795)	ND	8	ND	**≥64**	ND	**≥64**	**≥64**	**≥16**	ND	ND	0.5	≤1	4	0.5	**160**	≤1	0.25	2	ND
	*E. coli* J53 (pNDM-40)	**≥32**	**≥32**	64	**≥64**	**≥64**	**≥64**	8	**≥16**	**≥8**	≤1	≤0.25	≤1	4	≤0.25	≤20	≤1	0.25	2	**>128**
	*E. coli* EC600 (pNDM-40)	**≥32**	**≥32**	**≥128**	**≥64**	**≥64**	**≥64**	**16**	**≥16**	**≥8**	≤1	≤0.25	≤1	≤2	0.5	≤20	≤1	0.125	2	**>128**

Numbers in bold mean resistance. ND, not detected. AMP, ampicillin; SAM, ampicillin/sulbactam; TZP, piperacillin/tazobactam; CAZ, ceftazidime; CTT, cefotetan; CRO, ceftriaxone; FEP, cefepime; IMP, imipenem; ERP, ertapenem; ATM, aztreonam; CIP, ciprofloxacin; GN, gentamicin; AK, amikacin; LEV, levofloxacin; SXT, sulfamethoxazole/trimethoprim; TOB, tobramycin; TGC, tigecycline; CO, colistin; CZA, ceftazidime/avibactam.

### Location of carbapenem-resistant genes and its transferability of plasmid

**Figure 1:**
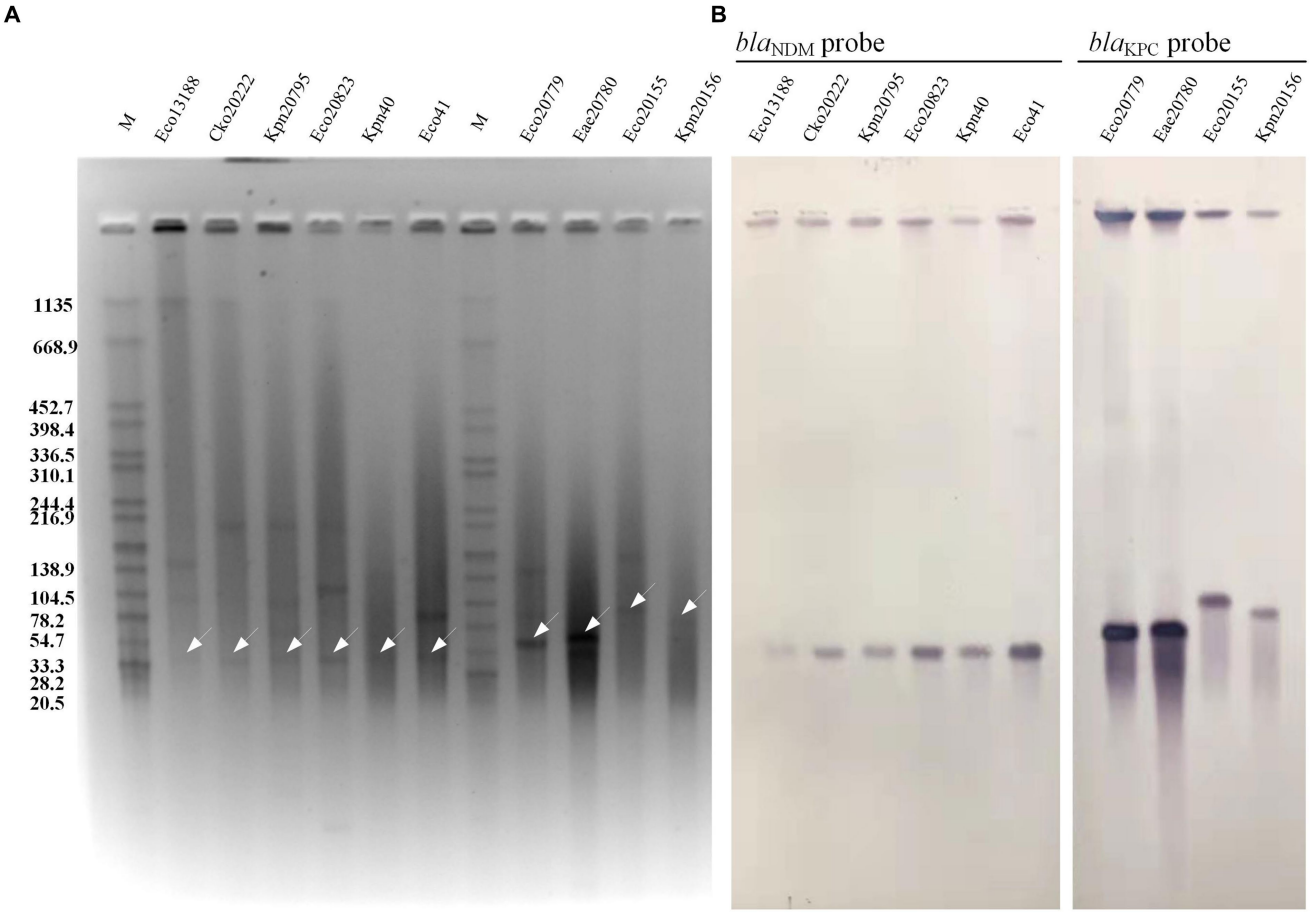
S1 PFGE and Southern blot hybridization of 10 CRE isolates. **(A)** S1 PFGE of the 10 CRE isolates. **(B)** Southern blot hybridization of probe *bla*_NDM_ and *bla*_KPC_. The white arrows showed the positive bands of plasmid hybridized with *bla*_NDM_ probe or *bla*_KPC_ probe. M, the *Salmonella* serotype Braenderup strain H9812 was used as a molecular marker.

As depicted in Figure 1, the six CRE isolates from P1 (Eco13188 & Cko20222), P4 (Kpn20795 & Ecl20823), and P5 (Kpn40 & Eco41) exhibited a positive signal for the *bla*_NDM_ probe, confirming the presence of *bla*_NDM_ gene on plasmids. The six *bla*_NDM_-carrying plasmids had nearly the same size within the range of 33.3 kb to 54.7 kb. The remaining four CRE isolates from P2 (Eco20779 & Eae20780) and P3 (Eco20155 & Kpn20156) showed a *bla*_KPC_ signal, indicating the carriage of *bla*_KPC_ gene on plasmids. The two *bla*_KPC_-carrying plasmids of P2 had the same size within the range of 54.7 kb ~78.2 kb, while the other two plasmids of P3 had obvious size differences.

The subsequent filter mating experiment showed that *bla*_KPC_-carrying isolates Eco20779 (*E. coli*) and Eae20780 (*K. aerogenes*) from P2 successfully transconjugated plasmids to recipients of other species, while Eco20155 (*E. coli*) and Kpn20156 (*K. pneumoniae*) from P3 failed to transfer plasmids to the selected recipients (Table 2, Transconjugant part). Specifically, Eco20779 and Eae20780 both transferred their KPC plasmids to *K. pneumoniae* ATCC13883 and *A. baumannii* ATCC17978, while Eae20780 transferred to *E. coli* J53 and *E. coli* EC600 in addition. The *bla*_NDM_-carrying isolates Kpn20795 (*K. pneumoniae*) from P4 successfully transferred its NDM plasmid to *E. coli* J53, *E. coli* EC600, and *A. baumannii* ATCC17978. And Kpn40 (*K. pneumoniae*) from P5 successfully transferred to *E. coli* J53, *E. coli* EC600. Eco13188 (*E. coli*) from P1 successfully transferred to *K. pneumoniae* ATCC13883.

Consequently, a total of 12 transconjugants were obtained from 5 CRE isolates, each from one patient. And all of the 12 transconjugants were resistant to imipenem and most β-lactams (Table 2, Transconjugant part), which were consistent with the phenotypes of the donors. S1-PFGE and southern blot analysis of the 12 transconjugants and donors confirmed the presence of *bla*_NDM_ or *bla*_KPC_-carrying plasmids (Supplementary Figure S1). Although not all the 10 CRE isolates successfully transferred the plasmid, the results still provide evidence that plasmids-carrying carbapenemases can be transferred interspecies.

### Genetic context of *bla*_NDM_ and *bla*_KPC_-carrying plasmids

Whole genome sequencing was conducted to investigate the genetic background of CRE isolates, and information of six complete genomes was shown in Table 3. Each genome contained sequences of chromosome and several plasmids, and the carbapenemase-carrying plasmids belonged to different plasmid incompatibility (Inc) types, mainly IncX3 and IncN.

**Table 3:** Genomic information of the six isolates.

Name	NCBI accession number	Location	Length (bp)	Plasmid incompatibility	Acquired resistance genes	Virulence genes
Eco13188	SAMN26149753	Chromosome	3,009,277	NA	ND	*gad*/*iss*/*ompT*/*terC*/*yfcV*
		Plasmid 1	1,826,733	ND	ND	*gad*/*lpfA*/*papC*
		Plasmid 2	210,076	ND	ND	*terC*
		Plasmid 3	157,982	IncFIB	*aadA2*/*aph(3′)-Ia*/*aph(4)-Ia*/*aac(3)-IV*/*bla*_CTX-M-14_/*bla*_TEM-1B_/*qacE*/*sitABCD*/*fosA3*/*mph(A)*/*floR*/*sul2*/*dfrA12*	*cma*/*cvaC*/*hlyF /iroN/iss/iucC/iutA/ompT/sitA/traT*
		Plasmid 4	112,005	IncFIB	ND	ND
		Plasmid 5	85,994	ND	ND	ND
		Plasmid 6	46,161	IncX3	*bla*_NDM-5_	ND
Eco20779	SAMN26149754	Chromosome	4,927,748	NA	*fosA7/mdf(A)*	*gad/iss/lpfA/terC*
		Plasmid 1	151,920	IncFIB	*aph(6)-Id/aac(3)-IId/aadA5/aph(3″)-Ib/bla*_CTX-M-65_ */qacE/sitABCD/mph(A)/sul1/sul2/tet(A)/dfrA17*	*cib/cma/cvaC/hlyF/iroN/iss/iucC/iutA/ompT/sitA/traT*
		Plasmid 2	94,723	IncY	ND	ND
		Plasmid 3	66,270	IncN	*aac(6′)-Ib3/bla*_KPC-2_ */bla*_TEM-1B_ */bla*_CTX-M-3_ */qnrS1/ARR-3/dfrA14*	ND
		Plasmid 4	55,471	IncI2	ND	ND
Eco20155	SAMN26149755	Chromosome	5,049,693	NA	*sitABCD/mdf(A)*	*air/chuA/eilA/gad/iss/kpsE/kpsMII_K5/lpfA/ompT/sitA/terC/yfcV*
		Plasmid 1	169,549	IncFIB	*aph(6)-Id/aph(3″)-Ib/bla*_TEM-1B_ */sitABCD/sul2/tet(A)*	*cvaC/etsC/hlyF/iroN/iss/iucC/iutA/mchF/ompT/sitA/traT/tsh*
		Plasmid 2	105,552	ND	*bla*_KPC-2_ */qnrS1*	*traT*
Kpn20156	SAMN26149756	Chromosome	5,365,552	NA	*aac(3)-IId/bla_SHV-28_/OqxA/OqxB/fosA*	*fyuA/irp2/iutA*
		Plasmid 1	201,928	IncFIB	*aac(6′)-Ib-cr/bla*_OXA-1_ */bla*_TEM-1B_ */bla*_CTX-M-15_ */mph(A)/catB3/tet(A)*	*traT*
		Plasmid 2	94,053	ND	*bla*_KPC-2_	*traT*
Kpn20795	SAMN26149757	Chromosome	5,001,340	NA	*bla*_SHV-33_ */OqxA/OqxB/fosA*	*iutA*
		Plasmid 1	217,814	IncFIB	ND	ND
		Plasmid 2	110,124	ND	ND	ND
		Plasmid 3	77,552	IncR	ND	ND
		Plasmid 4	46,224	IncX3	*bla*_NDM-1_	ND
Kpn40	SAMN26149758	Chromosome	5,197,000	NA	*bla*_SHV-155_ */OqxA/OqxB/fosA*	*iutA*
		Plasmid 1	144,306	IncFIB	ND	*traT*
		Plasmid 2	46,161	IncX3	*bla*_NDM-5_	ND

The plasmid incompatibility, acquired resistance genes and virulence genes were analyzed by software PlasmidFinder, ResFinder and VirulenceFinder on the website of Center of the Genomics Epidemiology. NA, not available; ND, not detected.

**Figure 2:**
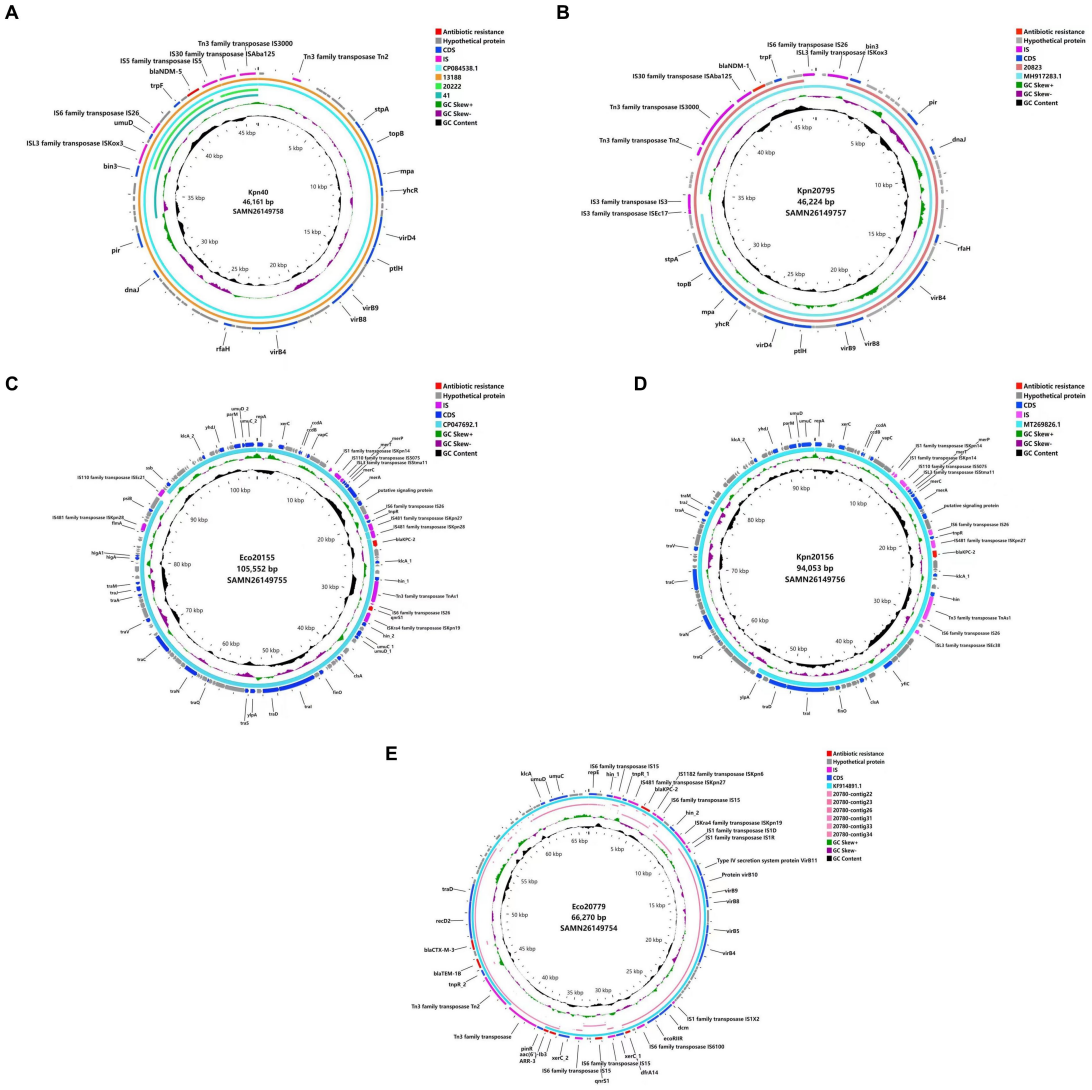
Plasmid comparison based on WGS. **(A)** WGS of Kpn40 plasmid versus reference sequence CP084538.1, Eco13188, Cko20222, and Eco41. **(B)** WGS of Kpn20795 plasmid versus reference sequence MH917283.1, and Ecl20823. **(C)** WGS of Eco20155 plasmid versus reference sequence CP047692.1. **(D)** WGS of Kpn20156 plasmid versus reference sequence MT269826.1. **(E)** WGS of Eco20779 plasmid versus reference sequence KF914891.1, and Eae20780 contig22,23,26,31,33,34. Different colors represent various genetic types. 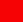, antibiotic resistance; 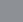, hypothetical protein; 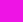, IS; 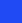, CDS; 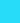, reference sequence; 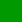, GC Skew+; 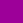, GC Skew−; 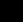, GC content; 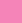 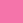 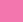 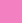 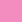 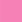, Eae20780 contig22, 23, 26, 31, 33, 34; 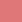, Ecl20823; 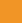, Eco13188; 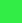, Cko20222; 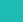, Eco41.

Genomic analysis of plasmids revealed the possible horizontal gene transfer (HGT) approach of carbapenemases (Figure 2). The four *bla*_NDM-5_-carrying plasmids from Eco13188, Cko20222, Kpn40 and Eco41 were almost identical in size, and had nearly the same surrounding context. Two *bla*_NDM-1_-carrying plasmids from Kpn20795 and Ecl20823 were almost identical in size and surrounding context of *bla*_NDM-1_, varying in some IS elements. The context of *bla*_NDM-1_-carrying plasmids were similar to the *bla*_NDM-5_ plasmid. The *bla*_KPC-2_-harboring plasmids from Eco20779 and Eae20780 were not the very same as the plasmid sequences of Eae20780 were assembled to several small contigs, while the main context and the surrounding context of *bla*_KPC-2_ was overlapped. Besides *bla*_KPC-2_, the plasmids also contained *bla*_CTX-M-3_ and *bla*_TEM-1_, flanked by *tnpR* gene and Tn3 family transposase, constituting a classical Tn3 transposon. The other two *bla*_KPC-2_-harboring plasmids from Eco20155 and Kpn20156 varied in sizes, but they shared a similar context of flanking region by *bla*_KPC-2_, which was a *ca.*10 kb IS26 associated HGT element. The main difference between the two elements was an addition of ISKpn28 fragment downstream ISKpn27 in plasmid of Eco20155. The carbapenemase gene contexts suggested the horizontal gene transfer capacity of carbapenemase-carrying plasmids.

## Discussion

Colonization of CRE in the gut is an asymptomatic infection (Nordmann and Poirel, 2019) that poses a potential risk to immunocompromised patients, such as those with hematological malignancies or undergoing chemotherapy for cancer (Tancrede and Andremont, 1985; Trecarichi et al., 2012; Shimasaki et al., 2019). This can lead to bacteremia through bacterial translocation, contributing to high mortality rates in these patient populations. In our previous screening project, carbapenem-resistant *K. pneumoniae* (CRKP) and carbapenem-resistant *E. coli* (CREC) were the predominant species in patients’ gut, and the co-existence of two CRE species in one patient was uncommon. In this study, we isolated 5 pairs of co-existent CRE species from 5 severely ill patients. During their treatment, the frequent use of carbapenem, tigecycline, or piperacillin/tazobactam posed a high risk for CRE proliferation due to microbiota dysbiosis (Harris et al., 2021). Studies have shown that antibiotic administration can lead to a significant increase in CRKP populations, owing to microbiota dysbiosis induced by antibiotic stress (Rooney et al., 2019). Under antibiotic stress, gut microbiota dysbiosis makes CRE take a competitive advantage and obtain a large number of nutrients, which promotes CRE growth and colonization (Rivera-Chavez et al., 2017). Also, the metabolites of normal intestinal flora can inhibit the growth of pathogens, while the destruction of intestinal flora reduces the metabolites, which ultimately leads to the massive growth and colonization of pathogens (Jacobson et al., 2018; McDonald et al., 2018; Sorbara et al., 2019). Therefore, it is necessary to screen for intestinal CRE in immunocompromised patients, evaluate the risk of CRE colonization, and maintain the stability of intestinal flora, both during the treatment and upon admission.

Plasmids are mobile genetic elements that play a crucial role in horizontal gene transfer. Conjugation is the primary mechanism through which plasmids facilitate HGT, with approximately 14% of plasmids being conjugative (Smillie et al., 2010). Previous research has predominantly focused on demonstrating plasmid-mediated transfer of antibiotic resistance genes *in vitro*. However, our study provides evidence of *in vivo* HGT of plasmids carrying antibiotic resistance genes.

As previously mentioned, we collected 10 CRE isolates from 5 patients, with each patient harboring 2 species. Through genome sequencing and conjugation experiments, we confirmed that *bla*_KPC_ and *bla*_NDM_ were located on plasmids. The 6 *bla*_NDM_-carrying plasmids from patients P1, P4, and P5 were all IncX3 type conjugative plasmids containing a T4SS system, which enhances the efficiency of plasmid conjugation (Smillie et al., 2010; Costa et al., 2021). Furthermore, the genomic context of plasmids from patients P1 and P5 (*bla*_NDM-5_) and P4 (*bla*_NDM-1_) were largely similar, differing only in a few IS insertions. Liu et al. reported that the backbone of IncX3 plasmids has remained highly conserved over 10 years, while the genetic context of *bla*_NDM_ has diversified into 5 subtypes, indicating the potent ability of *bla*_NDM_ genetic context (IS and transposon structures) to spread across different species (Liu et al., 2021). A recent study also confirmed that an IncX3 plasmid carrying *bla*_NDM_ was present in 47 different isolates of Enterobacteriales species in Hong Kong (Wang et al., 2018).

The IncX3 plasmid, which typically has a narrow spectrum of Enterobacteriales, was found to carry the *bla*_NDM-1_ gene in P4-Kpn20795, a strain of *K. pneumoniae*. This plasmid was successfully transferred to *A. baumannii* ATCC17978, indicating the potential for horizontal gene transfer between different species. The presence of ISAba125 in the context of *bla*_NDM_ suggests its involvement in the mobilization of *bla*_NDM_, a phenomenon that has been observed in *Acinetobacter* spp. as well (Nigro et al., 2011). Additionally, the *bla*_NDM-5_-carrying isolates from P1 were collected in a time sequence, with P1-Cko20222 being collected 3 days later than P1-Eco13188. This time interval indicates the possibility of *in vivo* transmission from *E. coli* to *C. koseri*, highlighting the occurrence of interspecies plasmid transfer within the gut. A similar *in vivo* transfer was reported by H. L. Nielsen et al., where a *bla*_NDM-1_-carrying IncN2 plasmid was identified in *Salmonella Kottbus* from the fecal sample of an individual, and was also found in *E. coli* and *Citrobacter freundii* in the same patient, suggesting a transfer between species (Nielsen et al., 2021).

The *bla*_KPC_ plasmids from P2, identified as IncN type, were found to be identical and capable of conjugating to both *K. pneumoniae* ATCC13883 and *A. baumannii* ATCC17978, demonstrating their ability for interspecies transfer. Previous studies have also provided evidence of interspecies transfer of *bla*_KPC_-carrying plasmids, such as the transmission of KPC-2 carbapenemase between *C. freundii*, *K. pneumoniae*, *E. coli*, and *Morganella morganii* during a patient’s long-term hospital stay (Polcarova et al., 2019). The IncN type plasmids are known for their broad-host range and ability to carry various resistance genes, including extended-spectrum β-lactams, quinolones, and aminoglycosides (Rozwandowicz et al., 2018). In addition to *bla*_KPC_, the P2 plasmids also carried *bla*_CTX-M-3_ and *bla*_TEM-1_, with *bla*_CTX-M_ often being associated with IncN plasmids in *E. coli*. On the other hand, the *bla*_KPC_ plasmids from P3 did not belong to any Inc. type according to PlasmidFinder analysis and failed in conjugation. Despite this, a manual inspection of the P3 plasmids revealed the presence of the *repA* gene and IncF plasmid conjugative transfer associated *tra* genes, although this could not explain the failure of conjugation.

## Conclusion

In conclusion, our study has demonstrated that the *bla*_KPC_ and *bla*_NDM_ carbapenemases of the 10 CRE isolates are located on plasmids and can be transferred interspecies through horizontal gene transfer in human intestinal environment. CRE intestinal colonization brings the risk of carbapenem resistance genes spreading in the host, so appropriate intestinal CRE screening and colonization prevention are necessary.

## Data availability statement

The datasets presented in this study can be found in online repositories. The names of the repository/repositories and accession number(s) can be found at: https://www.ncbi.nlm.nih.gov/, PRJNA809193.

## Ethics statement

This work was approved by Ethics Committee of Sir Run Run Shaw Hospital (no. 20200831-36).

## Author contributions

JJ: Writing – review & editing, Writing – original draft. YZ: Writing – review & editing, Project administration, Methodology. FZ: Writing – review & editing, Project administration, Methodology. JiZ: Writing – review & editing, Formal Analysis. BY: Writing – review & editing, Project administration, Methodology. MZ: Writing – review & editing, Formal Analysis. YY: Writing – review & editing, Conceptualization. JuZ: Writing – review & editing, Conceptualization. YF: Writing – review & editing, Conceptualization.

## Footnotes

^1^https://proksee.ca/projects/new

^2^http://www.genomicepidemiology.org/
