# Supplementary material for: Co-colonization of different species harboring KPC or NDM carbapenemase in the same host gut: insight of resistance evolution by horizontal gene transfer
Source: Front Microbiol. 2024 Jun 14;15:1416454. doi: 10.3389/fmicb.2024.1416454 (PMC11211256; doi:10.3389/fmicb.2024.1416454)
Supplement: Supplementary file 1 [file Data_Sheet_1.PDF]

Supplemental TABLE1. Combination of donor and recipient and antibiotic selection in filter mating experiment (μg/mL)

Donor cell		Recipient cell (Antibiotic phenotype)			
Isolate	Antibiotic phenotype	*E. coli* J53 (NaN3^R, AMP^S)	*E. coli* EC600 (RIF^R, AMP^S)	*K. pneumoniae* ATCC13883 (RIF^R, MEM^S)	*A. baumannii* ATCC17978 (RIF^R, MEM^S)
Eco13188	NaN3^S, RIF^S, MEM^R	NA	NA	**RIF 500+MEM 4**	RIF 500+MEM 4
Cko20222	NaN3^S, RIF^R, MEM^R	NaN3 120+MEM 4	NA	NA	NA
Eco20779	NaN3^S, RIF^S, MEM^R	NA	NA	**RIF 500+MEM 4**	**RIF 500+MEM 4**
Eae20780	NaN3^S, RIF^S, MEM^R	**NaN3 120+MEM 4**	**RIF 800+MEM 4**	**RIF 800+MEM 4**	**RIF 800+MEM 4**
Eco20155	NaN3^S, RIF^S, MEM^R	NA	NA	RIF 500+MEM 2	RIF 500+MEM 2
Kpn20156	NaN3^S, RIF^S, MEM^R	NaN3 120+AMP 200	RIF 500+AMP 200	NA	RIF 500+MEM 2
Kpn20795	NaN3^S, RIF^S, MEM^R	**NaN3 120+AMP 200**	**RIF 500+AMP 200**	NA	**RIF 500+MEM 4**
Ecl20823	NaN3^S, RIF^R, MEM^R	NaN3 120+MEM 4	NA	NA	NA
Kpn40	NaN3^S, RIF^S, MEM^R	**NaN3 120+AMP 200**	**RIF 500+AMP 200**	NA	RIF 500+MEM 4
Eco41	NaN3^S, RIF^S, MEM^R	NA	NA	RIF 800+MEM 4	RIF 800+MEM 4

NA, not available because of the same species between the donor and the recipient or antibiotic selection. AMP, ampicillin; NaN3, Sodium azide; MEM, meropenem; RIF, rifampicin. S, susceptible; R, resistance.

Antibiotic combinations in bold are successful conjugations.

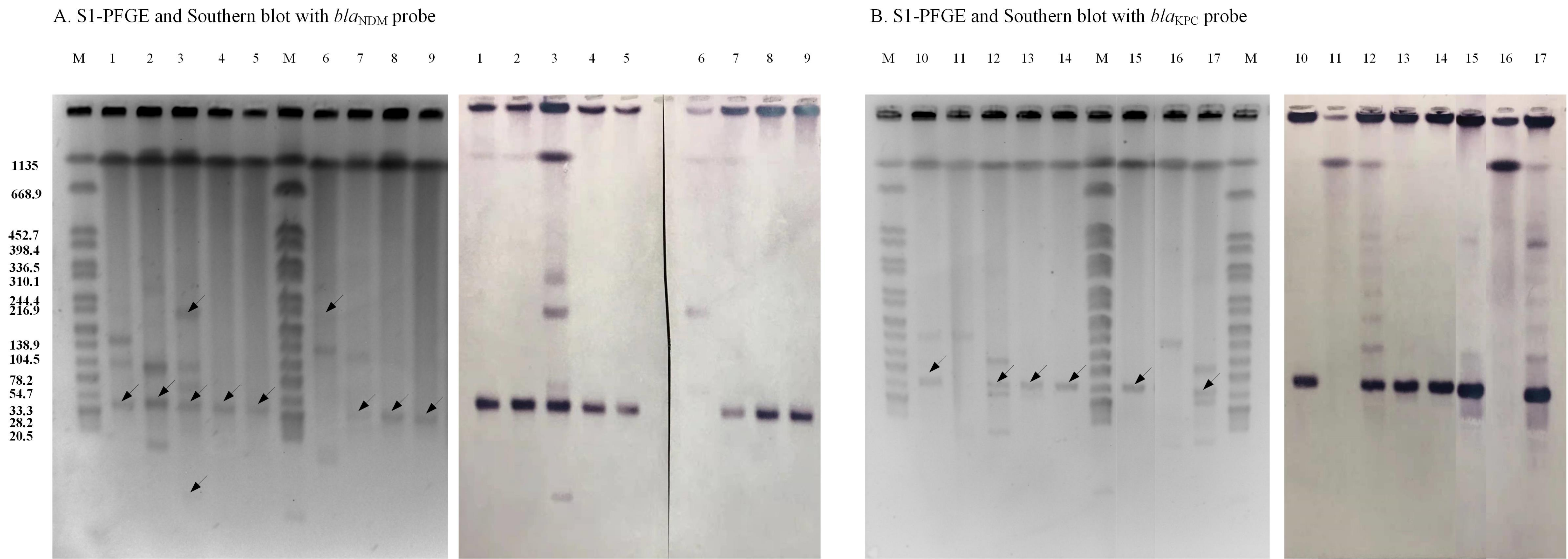
A. S1-PFGE and Southern blot with bla_{NDM} probe
B. S1-PFGE and Southern blot with bla_{KPC} probe
M 1 2 3 4 5 M 6 7 8 9
1 2 3 4 5 6 7 8 9
M 10 11 12 13 14 M 15 16 17 M
10 11 12 13 14 15 16 17
1135
668.9
452.7
398.4
336.5
310.1
244.4
216.9
138.9
104.5
78.2
54.7
33.3
28.2
20.5

Supplemental FIGURE1. S1-PFGE and Southern blot hybridization of donors and transconjugants.
(A) S1-PFGE and southern blot with *bla*$_{NDM}$ probe. (B) S1-PFGE and southern blot with *bla*$_{KPC}$ probe. 1, Eco13188; 2, *K. pneumoniae* ATCC13883 (pNDM-13188); 3, Kpn20795; 4, *E. coli* J53 (pNDM-20795); 5, *E. coli* EC600 (pNDM-20795); 6, *A. baumannii* ATCC17978 (pNDM-20795); 7, Kpn40; 8, *E. coli* J53 (pNDM-40); 9, *E. coli* EC600 (pNDM-40); 10, Eco20779; 11, *K. pneumoniae* ATCC13883(pKPC-20779); 12, *A. baumannii* ATCC17978 (pKPC-20779); 13, Eae20780; 14, *E. coli* J53 (pKPC-20780); 15, *E. coli* EC600(pKPC-20780);16, *A. baumannii* ATCC17978 (pKPC-20780); 17, *K. pneumoniae* ATCC13883 (pKPC-20780). The black arrows showed the positive bands of plasmid hybridized with *bla*$_{NDM}$ probe or *bla*$_{KPC}$ probe. M, The Salmonella serotype Braenderup strain H9812 was used as a molecular marker.
